# Costs and Cost-Effectiveness of *Plasmodium vivax* Control

**DOI:** 10.4269/ajtmh.16-0182

**Published:** 2016-12-28

**Authors:** Michael T. White, Shunmay Yeung, Edith Patouillard, Richard Cibulskis

**Affiliations:** 1 MRC Centre for Outbreak Analysis and Modelling, Department of Infectious Disease Epidemiology, Imperial College London, London, United Kingdom.; 2 Department of Global Health and Development, London School of Hygiene and Tropical Medicine, London, United Kingdom.; 3 Global Malaria Programme, World Health Organization, Geneva, Switzerland.; 4 Swiss Tropical and Public Health Institute, Basel, Switzerland.; 5 Universität Basel, Basel, Switzerland.

## Abstract

The continued success of efforts to reduce the global malaria burden will require sustained funding for interventions specifically targeting *Plasmodium vivax*. The optimal use of limited financial resources necessitates cost and cost-effectiveness analyses of strategies for diagnosing and treating *P. vivax* and vector control tools. Herein, we review the existing published evidence on the costs and cost-effectiveness of interventions for controlling *P. vivax*, identifying nine studies focused on diagnosis and treatment and seven studies focused on vector control. Although many of the results from the much more extensive *P. falciparum* literature can be applied to *P. vivax*, it is not always possible to extrapolate results from *P. falciparum*–specific cost-effectiveness analyses. Notably, there is a need for additional studies to evaluate the potential cost-effectiveness of radical cure with primaquine for the prevention of *P. vivax* relapses with glucose-6-phosphate dehydrogenase testing.

## Introduction

*Plasmodium vivax* is the most widely distributed species of malaria across the world, with almost 3 billion people at risk and an estimated 13.8 (10.3–18.4) million clinical cases every year, mostly in Asia, the Horn of Africa, and South America.^1^–^4^ Almost half of all malaria cases outside Africa are attributable to *P. vivax*.^5^ The scale of the public health burden has been highlighted by the increasing evidence for the magnitude of severe and fatal disease caused by *P. vivax*.^3^ Over the past decade, the concerted scale-up of malaria control efforts, in particular long-lasting insecticidal nets (LLINs) and artemisinin combination therapies (ACT), has resulted in significant reductions in the burden of malaria, but with an increase in the ratio of *P. vivax* to *Plasmodium falciparum* cases in many areas where the two species coexist.^6^,^7^ In most countries in the malaria preelimination or elimination phases, *P. vivax* is the dominant species.^5^ It is likely that continued progress may require increased financing for interventions specifically directed toward the *P. vivax* hypnozoite reservoir and innovative vector control tools along with an improved understanding of the relationship between costs and public health benefit.

Much of the theoretical and empirical work on the costs and cost-effectiveness of malaria control interventions has focused on *P. falciparum* malaria,^8^,^9^ due to its high prevalence in sub-Saharan Africa where the majority of the burden of cases and deaths from malaria occur. The costs and cost-effectiveness of interventions for controlling *P. falciparum* are usually analyzed in isolation without the need to consider detailed interactions with other *Plasmodium* species, most notably *P. vivax*.^8^–^10^ In contrast, studies of *P. vivax* are often undertaken in areas that are coendemic with *P. falciparum*, thus requiring costs and benefits to be apportioned between the two species of malaria. Although some of the costs from studies focusing on *P. falciparum* can be used to estimate the costs related to *P. vivax* control, they cannot simply be extrapolated from an African setting to an Asian, western Pacific, or South American setting, nor can measures of effectiveness be extrapolated from *P. falciparum* to *P. vivax*. Furthermore, *P. falciparum* focused studies provide limited insight for *P. vivax*–specific interventions that target the hypnozoite reservoir.

The biology and epidemiology of *P. vivax* present a number of complicating factors that must be accounted for in studies for the evaluation of the costs and cost-effectiveness of certain interventions, most notably relapses due to hypnozoites, which are only killed by 8-aminoquinoline drugs^11^; and the risk of hemolysis in glucose-6-phosphate dehydrogenase (G6PD)–deficient patients after treatment with 8-aminoquinoline therapies.^12^ The impact of these phenomena on the effectiveness of *P. vivax* control interventions (vector control or treatment with antimalarials) has been documented in some cases,^13^ but the integration of the effects of relapses and hemolysis into studies of cost-effectiveness remains an ongoing challenge.

Herein, we review the evidence from published studies on the costs and cost-effectiveness of interventions for controlling *P. vivax*, and consider the economic consequences of controlling or failing to control *P. vivax* malaria.

## Measuring Costs, Effects, and Cost-Effectiveness of Interventions

When evaluating the cost of interventions, the perspective taken can be that of the health-care provider, the patient, or both (the societal cost).^14^ Depending on the intervention, provider costs include the cost of consumables such as insecticide-treated bed nets (ITNs), drugs and rapid diagnostic tests (RDTs), and the costs associated with implementation. These can include start-up capital costs (such as buildings and vehicles), recurrent costs (such as personnel and overheads), and sometimes costs of starting up new interventions (such as training) or implementing supportive interventions (such as community sensitisation). Extending to a societal perspective requires inclusion of direct household costs of illness (such as travel, drugs, and consultation costs) which vary greatly and can be difficult to estimate.^15^ Costs can be expressed as the total costs of an intervention for a given area or population, or as cost per unit delivered or person protected. Furthermore, there is a distinction between financial and economic costs. Financial and economic costs reflect the unit cost of an intervention and the resources required for its delivery in terms of the actual expenditures incurred. The economic costs capture the opportunity cost of all resources used to provide an intervention, whether or not they incur a financial expenditure.

The cost-effectiveness of an intervention is the ratio of the cost to a relevant measure of its effect, and is often compared with a counterfactual of “doing nothing” or else the cost-effectiveness of an existing intervention (referred to as incremental cost-effectiveness when a comparison is made). The choice of outcome measure depends on the intervention and the perspective taken; for example, numbers correctly treated, numbers cured, number of cases and deaths averted, or the number of deaths or disability-adjusted life years (DALYs) averted. In particular, multiple effectiveness metrics can be defined for each intervention. Data on effects can come from routine health system data (e.g., number of clinical malaria cases treated and number of malaria-related deaths in the public health system) or research studies. However, studies measuring impact on transmission or mortality can be demanding to undertake, and therefore, economic and mathematical modeling is often used to predict the impact of interventions or packages of interventions on outcomes.^16^,^17^

A checklist for critical appraisal of health economic evaluation studies is provided by Drummond's criteria,^14^,^18^ which appraises whether costs and effects are adequately evaluated and compared with competing alternatives. An updated set of guiding principles, methodological specifications, and reporting standards to support cost-effectiveness evaluations of health interventions has recently been compiled by The Bill & Melinda Gates Foundation, the National Institute for Health and Care Excellence and partners, and presented in the Gates Reference Case.^19^ The reference case outlines a number of principles that should be considered during economic evaluations, including transparency, uncertainty, equity, time horizons, and appropriate measures of health outcomes. These principles provide a contemporary gold standard for the economic evaluation of health interventions.

## Models for Estimating the Cost-Effectiveness of *P. vivax* Interventions

**Figure 1.**
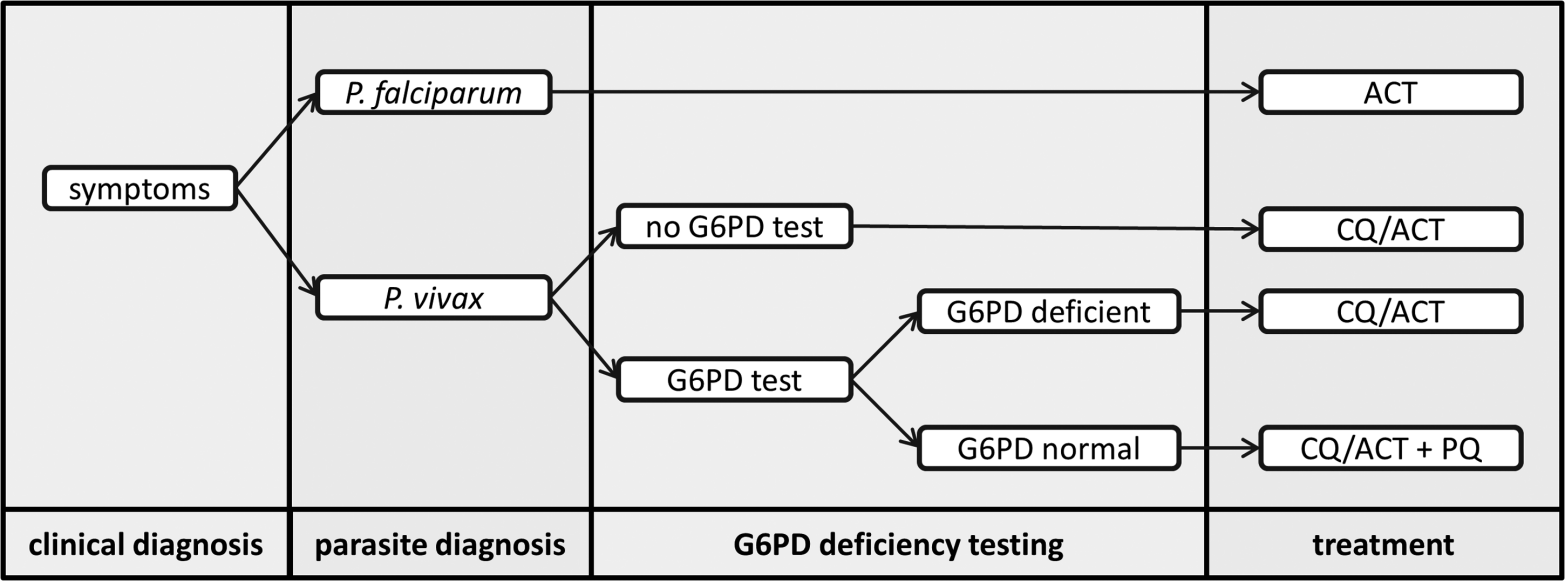
Schematic of a decision tree model for cost-effectiveness analysis in a coendemic *Plasmodium falciparum* and *Plasmodium vivax* setting, with endpoints of correctly diagnosed and treated cases.

Cost-effectiveness analyses frequently utilize models to combine data with realistic assumptions such as diagnostic performance or treatment success rates. Decision tree models which track the progress of a patient through a treatment pathway are frequently used as they allow uncertainty to be handled robustly. They have been widely applied to the diagnosis and treatment of both *P. falciparum* and *P. vivax*.^20^–^22^ Figure 1

shows an example of a probabilistic decision tree model suitable for analyzing data from a study of treatment and diagnosis in a *P. vivax* and *P. falciparum* coendemic setting. At each node in the tree, the probability of progressing to the next node will be determined by the collected data or prior knowledge of the properties of the diagnostic tool (e.g., sensitivity and specificity). Running repeated simulations with the probabilities at each node varied within a range determined by a sensitivity analysis allows the uncertainty in estimates of costs and cost-effectiveness to be captured. Decision tree models can also be used to assess the feasibility of alternative strategies such as providing primaquine to all malaria cases in areas where *P. falciparum* infection is a good predictor of future *P. vivax* relapses.^13^,^23^

Markov models describe a number of states and likelihoods of moving from one state to another. Unlike decision tree models, Markov models allow loops back into previous states and are therefore often used in chronic diseases or relapsing diseases.^24^ Existing economic models of *P. vivax* have been based on decision trees^20^; however, the relapsing nature of the disease lends itself to a Markov model.

## Overview of Existing Work on Cost-Effectiveness of *P. vivax* Interventions

Herein, we present an overview of the costs and cost-effectiveness of controlling *P. vivax* malaria based on the information available in the publicly available literature. A review of the published literature on the costs and cost-effectiveness of *P. vivax* control was conducted using the online database PubMed. The search term used was “vivax” and “cost” or “economic.” This was supplemented by reviews of the reference lists of relevant published papers. All studies reviewed focused either on diagnosis, treatment, or vector control. The results are presented in Table 1 (diagnosis and treatment) and Table 2 (vector control). All the identified studies were based on intervention trials, except for one study on the cost-effectiveness of diagnosis and treatment in the Brazilian Amazon.^32^ The number of studies on *P. vivax* was much less than the substantial literature on *P. falciparum*.^10^ For example, when van Vugt and others^41^ undertook a systematic review of the cost-effectiveness of malaria treatment and prophylaxis, they identified 17 studies, only two of which included estimates of the cost-effectiveness of testing or treating *P. vivax*. Studies scored highly when graded against Drummond's criteria^18^ (Table 1, Table 2, and Supplemental Table 1), indicating a good quality of evidence, particularly among studies published since 2000.

## Costs and Cost-Effectiveness of Diagnosis and Treatment

Episodes of *P. vivax* malaria can be treated using blood schizontocidal drugs such as chloroquine (CQ) (in areas free from resistance^42^) and ACTs.^43^ Blood schizontocidal drugs clear blood-stage parasites and reduce *P. vivax* associated morbidity, but do not affect the liver-stage hypnozoites responsible for relapses. There are few published studies on the costs and cost-effectiveness of case management specifically including *P. vivax.* Nine studies were identified (Table 1) with three focusing only on diagnostic testing. Only one study compared treatment with different blood schizontocidal regimens.^30^

In terms of diagnosis, the development and implementation of new diagnostic testing for *P. vivax* has lagged behind *P. falciparum*.^44^ Good-quality RDTs capable of detecting both species have become increasingly available in areas where the two species coexist.^45^ The cost of RDTs has been declining over time^46^ with pan-specific tests available in the range of 1–2 U.S. Dollars (USD).^27^ The cost-effectiveness studies that were surveyed used different costs and different measures of effectiveness such as “correctly treated” or “adequately diagnosed.” Two studies from different settings found RDTs to be a more cost-effectiveness option for diagnosis and treatment than microscopy (Table 1).^20^,^27^ In general, the cost-effectiveness of options for diagnosing *P. vivax* infections will depend on a number of factors including *P. vivax* prevalence, the operational accuracy of the different options (including presumptive and microscopy), cost of treatment, and provider adherence to the test result.^20^,^27^ A particularly important factor is the volume of patients: in a busy health center or hospital, microscopy may be very cost-effective per diagnosis, although there may be a trade-off in the time taken for diagnosis.

*Plasmodium* vivax infections can be treated with either CQ or ACTs.^28^,^47^ The decision to adopt treatment of *P. vivax* with ACTs (potentially as part of a unified treatment strategy) will depend on economic factors. The cost per full adult course of ACT has been estimated to be in the range 0.92–3.85 USD, compared with 0.07–0.10 USD for CQ.^43^ However, the cost of ACTs has been steadily declining.^48^ A study on the cost-effectiveness of treatment of uncomplicated malaria in children from a *P. vivax* and *P. falciparum* coendemic region of Papua New Guinea found that, despite the increased costs of ACTs, the cost per case of *P. vivax* treated was comparable for ACTs and CQ + sulfadoxine–pyrimethamine^30^ (Table 1), and dihydroartemisinin–piperaquine was found to be the most cost-effective option for *P. vivax* using 42-day efficacy as the outcome measure. Despite this finding, further evidence on the cost-effectiveness of unified treatment strategies is needed.

## Radical Cure and G6PD Deficiency Testing

Compared with assessing the cost-effectiveness of blood schizontocidal drugs, there are a number of additional challenges associated with providing radical cure with hypnozoitocidal therapy against liver-stage parasites, in particular the occurrence of relapses and the potential for hemolysis in G6PD-deficient patients treated with 8-aminoquinolines.^12^ Each relapse may cause a debilitating febrile illness with deepening risk of severe anemia or other complications associated with fatal outcomes, along with opportunities for continued transmission in the community.

Currently, primaquine, an 8-aminoquinoline, is the only drug currently available that effectively eliminates hypnozoites, resulting in radical cure and therefore with the potential to cause large reductions in morbidity and mortality. Primaquine is inexpensive, costing 0.15–0.60 USD per course.^49^–^51^ However, there are two major disadvantages to its use as a hypnozoitocidal drug: a standard regimen of 14 days resulting in poor adherence; and the potential to cause life-threatening hemolysis in patients with G6PD deficiency, an inherited disorder. Tafenoquine, another 8-aminoquinoline, is currently undergoing Phase 3 clinical trials, and is likely to be at least as efficacious as primaquine,^52^ but has a much longer half-life and will therefore only require a single dose. Although it is likely to be more expensive than primaquine, the single dose may provide better levels of adherence to a full therapeutic course. However, this promising new therapy also puts patients with G6PD deficiency at risk of potentially fatal hemolysis without the option of abandoning the treatment course midway. The problem of screening out vulnerable patients from receiving it will be a crucial task in terms of real-world access to the drug and its huge clinical and public health benefits.

Screening for G6PD deficiency is possible, but has previously been limited to higher level health facilities.^53^ A number of different point of care diagnostics are under development, with some experience of using them in the field.^54^,^55^ Costs per assay are in the range of 1.50–20.00 USD^23^; however, once implementation costs are included, the minimum cost per subject tested is unlikely to be less than 4.00 USD. It is possible that the production of currently available tests could be optimized so that the price becomes comparable to parasitological RDTs costing approximately 0.50 USD per test.^56^,^57^ Notably, the cost of widespread testing for G6PD deficiency may be less than the alternative of providing primaquine without testing and treating subsequent episodes of hemolysis. For example, in the Brazilian Amazon providing primaquine without prior G6PD testing to infected males may be associated with excess deaths, as well as an estimated annual cost to the health system of 4–5 million USD.^33^

The cost-effectiveness of 8-aminoquinoline treatment with and without prior G6PD deficiency testing will depend on a number of epidemiological, biological, behavioral, and drug factors. The effectiveness in different transmission settings is likely to be highest where the likelihood and frequency of relapses is highest and the proportion of cases attributable to new/reinfections relatively low. The effectiveness of the drug will be critically dependent on adherence which will depend on the choice of drug and dosing regimen. The risk of hemolysis after treatment will depend on a number of factors including^12^,^58^ 1) the overall prevalence of different variants of G6PD deficiency in the population and the susceptibility of the variant to hemolysis, 2) the gender of the patient, and 3) the dosing regimen of the 8-aminoquinoline. Finally, the incremental cost-effectiveness of G6PD deficiency testing will depend on the cost of the test, sensitivity, and specificity for correctly diagnosing individuals at significant risk of severe hemolysis, and uptake and quality of use under operational settings.

## Costs and Cost-Effectiveness of Vector Control

Targeting mosquitoes with vector control interventions can jointly combat *P. vivax* and *P. falciparum* malaria,^31^ as well as other vector-borne diseases.^59^ The potential impact of interventions will depend on the behavioral characteristics of local mosquito populations, such as the proportion of blood meals taken on humans and the proportion of exposure occurring indoors.^60^ In *P. falciparum*–endemic Africa, LLINs and indoor residual spraying (IRS) are the mainstays of vector control and are highly effective against *Anopheles gambiae*, the predominant vector which feeds almost exclusively on humans indoors during the hours of sleep. The wide range of vector species in *P. vivax*–endemic regions^61^ will result in substantial variation in the potential effectiveness of different vector control tools, with feeding on domesticated animals and early outdoor biting limiting the effectiveness of LLINs and IRS. Some interventions such as insecticide-treated hammocks and hammock nets have been specifically developed for outdoor use.^62^ These have been used in parts of Asia by populations at risk of *P. vivax* (e.g., forest workers, other highly mobile groups, and people living in traditional homes not suited to LLINs).^63^ Environmental control methods such as water drainage and larviciding can provide long-lasting cost-effective protection in areas with vulnerable local vector species.^64^

As has been reviewed elsewhere, there are few studies on the effectiveness of vector control interventions for reducing *P. vivax* transmission and morbidity^65^ (Hii and others, Vector control thematic review, AJTMH supplement 2016). There are even fewer studies on the cost-effectiveness of vector control against *P. vivax*, with none looking at *P. vivax* specifically. Table 2 presents the costs per case averted or person protected against malaria in six countries with both *P. vivax* and *P. falciparum*. Four studies focused on ITNs and IRS. Three studies found ITNs to be more cost-effective than IRS.^34^–^38^ One study modeled the cost-effectiveness of LLINs compared with the provision of early RDT diagnosis and treatment using community volunteers in Myanmar.^31^ Compared with no interventions, ITNs were estimated to avert one DALY for every 51 USD spent. When ITNs were implemented alongside early diagnosis and effective treatment (EDAET), the incremental cost per DALY averted compared with EDAET alone was estimated as 148 USD. It should be noted that these studies provide estimates of cost-effectiveness of interventions against all episodes of malaria and are not stratified according to numbers of *P. falciparum* and *P. vivax* cases.

A study on long-lasting insecticide-treated hammocks found them to prevent cases of malaria in individuals sleeping and working in forested areas.^37^ The cost per case of malaria averted (*P. vivax* or *P. falciparum*) was estimated at 126 USD. In addition to ITNs and IRS, individuals frequently protect themselves using methods such as mosquito coils, aerosol sprays, vaporizing mats, and repellents. Although these tools have been proven to reduce the number of mosquito bites, evidence of protection against malaria in programmatic settings is scarce.^66^ Annual household expenditure on these tools has been reported as 4–25 USD in Thailand,^67^ 2.04–19.20 USD in rural Indian areas, and 15.60–26.40 USD in urban Indian areas.^68^,^69^

## Procurement Costs

The costs informing the studies in Tables 1 and 2 are based on commodity prices during the year of the study. However, the global scale-up of malaria control over the past decade has led to substantial reductions in the cost of procuring drugs, tests, and LLINs.^70^ For example, procurement costs for LLINs in *P. vivax*–endemic countries can be obtained from The Global Fund's Price and Quality Reporting tool. The average cost of procuring an LLIN in *P. vivax*–endemic regions has been reported as approximately three USD, ranging from 1.65 to 5.80 USD. In addition to procurement costs, the cost of distribution must be accounted for, usually found to be in the range 1–4 USD per net.^10^ The total cost of LLIN procurement and delivery is therefore likely to range between 4 and 7 USD and vary across net types and products.

## The Cost of Not Controlling *P. vivax*

The socioeconomic burden of *P. vivax* depends on a number of factors, most notably the estimated annual number of clinical cases and *P. vivax*–associated deaths^1^; the contribution of *P.* vivax to chronic anemia and; the economic costs of treatment, borne by either the individual patient or the health system; and the economic cost to a society associated with absence from work or school. There are likely to be substantial indirect costs due to lost productivity—three studies found that symptomatic episodes of malaria in Asia resulted in the loss of 4–15 days of work or school by the affected patient.^71^–^73^ In addition to effects that can be quantified economically, *P.* vivax has many other negative consequences. Recurrent episodes of *P. vivax* are also likely to have an adverse impact on school performance in children, the economic cost of which is difficult to estimate.^74^,^75^ *Plasmodium vivax* episodes are expected to result in anemia, malnutrition, growth retardation, and stunting of development, all of which lead to societal direct costs including health-care costs from provider and patient perspectives and indirect costs such as impaired economic productivity in later life.^3^

As well as affecting households on a microeconomic level, malaria is also likely to have macroeconomic effects. However, the associations between malaria burden and macroeconomic measures such as gross domestic product (GDP) growth rate are challenging to estimate due to issues of causality—do people get malaria because they are poor, or are people poor because they get malaria?^76^ In an analysis of the association between malaria and GDP growth, Sachs and others found that in countries with intense malaria, GDP growth was reduced by 1.3% per year^77^,^78^; however, this study predominantly focused on *P. falciparum* malaria in sub-Saharan Africa, and while its conclusions may not be extrapolated to *P. vivax*, it is likely that *P. vivax* has a nonnegligible impact on economic development. Finally, there is evidence that socioeconomic development is an effective intervention against malaria.^79^ Increased economic development will lead to strengthening of health systems and better treatment, and economic empowerment of individuals to better cope with episodes of malaria. Globally significant social and economic trends such as urbanization^80^ and improved road and transport networks are all likely to reduce malaria burden.

## Discussion

Although there is an emerging consensus of the need to further reduce the burden of *P. vivax* malaria, particularly given the renewed enthusiasm for malaria elimination,^81^ evidence for the costs and cost-effectiveness of *P. vivax* control and elimination is lacking. An increased evidence base on the cost-effectiveness of *P. vivax* control is crucial to make the case for increased and sustained funding for malaria control, and to make the most efficient use of existing, limited resources.

Valid comparison between the results of different studies is hampered by variation in malaria transmission intensity between locations, differences in methodologies, and in how the results for costs, effects, and cost-effectiveness are expressed. Only one study attempted to compare the cost-effectiveness of vector control with diagnosis and treatment, an approach which is potentially useful for making resource allocation decisions.^31^ Furthermore, most cost-effectiveness studies are based on intervention trials, so estimates of cost-effectiveness must be generalized to larger programmatic settings. Expanding *P. vivax* control measures will probably lead to economies of scale^10^ where the cost per unit intervention decreases, but also more importantly, to economies of scope as *P. vivax* control measures are integrated alongside surveillance and control measures for *P. falciparum* and possibly other diseases. Although in practice, control strategies are often integrated across all *Plasmodium* species at the programmatic level, in theory, cost-effectiveness analyses often focus on a single species. Evaluating the cost-effectiveness of *P. vivax* control interventions without accounting for the additional effects on *P. falciparum* may lead to the benefits of effective control measures being undersold. As integrated malaria control strategies are rolled out, appropriate methods for evaluating the cost-effectiveness of intervention packages are badly needed.

A limitation of many economic models used for evaluation of the cost-effectiveness of *P. vivax* interventions is that they are static and only capture the benefit to the individual being protected or treated, and not the additional benefits accruing due to reductions in transmission. Accounting for changes due to reduced transmission requires a model of the transmission dynamics of the *Plasmodium* parasite between humans and mosquitoes. Although there are several examples of *P. falciparum* transmission models being applied to cost-effectiveness problems,^16^,^17^ the capacity for modeling *P. vivax* transmission dynamics is much more limited with few published models.^82^–^85^ Incorporating a model of *P. vivax* transmission into cost-effectiveness analyses would allow for the benefits of reduced transmission after treatment to be accounted for, in particular the reduction in relapses after radical cure with primaquine. An additional benefit of using transmission models is that they can account for mixes of interventions where effects may not be additive. For example, if ITNs and treatment programs incorporating primaquine are deployed simultaneously,^31^ the benefits of reducing community-level transmission and preventing relapses are not likely to be additive.

The adoption of a unified treatment strategy for *P. falciparum* and *P. vivax* provides an opportunity for further integration of malaria controls, as there are substantial clinical and logistical benefits to treating *P. vivax* with ACTs instead of CQ.^28^,^34^ The clinical benefits are compelling in regions where CQ resistance has been reported.^27^ The short half-life of artemisinin may lead to reduced efficacy of ACT treatment against *P. vivax* relapses; however, this effect may be mitigated by selection of a partner drug with a long half-life.^29^ In areas where *P. vivax* and *P. falciparum* are coendemic, a unified treatment strategy incorporating ACTs and primaquine with testing for G6PD deficiency may allow simplified treatment protocols.^86^

In order for cost-effectiveness analyses to be used appropriately, there are several key knowledge gaps that need to be addressed.^56^,^87^ First, the contribution of *P. vivax* infection to severe anemia and the probability of progression to episodes of severe malaria and mortality need to be better estimated.^3^,^5^ Second, the dynamics of *P. vivax* transmission need to be understood to correctly estimate the impact of control measures on incidence, prevalence, and morbidity.^83^ Third, the operational effectiveness of different primaquine treatment schedules needs to be evaluated. In some cases, directly observed treatment of primaquine may be affordable and result in significant increases in effectiveness in preventing relapses. Finally, better data are needed on the likelihood and severity of hemolysis in G6PD-deficient individuals and the ability of diagnostic tests to categorize patients into those who are and are not at significant risk of hemolysis.

Research into the cost-effectiveness of malaria control has predominantly focused on the evaluation of interventions for treatment and prevention in endemic regions. However, in the future, as malaria control programs transition from control to elimination, a corresponding shift in the research agenda will be required, with more focus on the costs of surveillance, prevention of reintroduction, and responses to malaria epidemics.^88^,^89^ This is particularly true in regions that can sustain *P. vivax* transmission where elimination efforts are likely to consist of a protracted surveillance campaign to detect and respond to infections arising from *P. vivax* relapses long after other species of malaria have been eliminated.

## Supplementary Material

Supplemental Table.

## Figures and Tables

**Table 1:** Studies of the costs and cost-effectiveness of diagnosing and treating *Plasmodium vivax*

Study area (year)	Perspective and scope	*P. vivax* cases (%)	Drummond's criteria	Intervention	Cost 2013 USD	Cost-effectiveness 2013 USD
Diagnosis						
Amazon, Brazil (2006)^20^	Provider	67	10/10	*Pf/Pv* RDT (OptiMal)	4.95 per test	5.94 per case adequately diagnosed
	Serv. incl.			Microscopy	Not provided	7.85 per case adequately diagnosed
				Microscopy vs. OptiMal		635.45 per additional case adequately diagnosed
Sri Lanka (2001–2002)^25^	Provider	70	8/10	ICT *Pf*/*Pv* assay (70% sensitivity)	4.93 per test	14.36–143.62 per *Pv* infection detected
	Serv. incl.			microscopy		0.74–7.35 per *Pv* infection detected
Manila, Palawan, Philippines (2009)^26^	Provider	NA	NA	RDT (private sector: Panbio, Parascreen, Parabank, Paraview)	4.13–13.03 per RDT	No effectiveness data
Diagnosis and treatment						
Afghanistan (provider) (2009–2012)^27^	Provider	≥ 90	10/10	Moderate transmission		
	Serv. incl.			*Pf*/*Pv* RDT (CareStart)	2.08 per RDT	2.82 per patient tested and treated
				Microscopy	2.05 per test by microscopy	2.82 per patient tested and treated
				Low transmission		
				*Pf/Pv* RDT (CareStart)	1.30 per RDT	2.06 per patient tested and treated
				Microscopy	8.32 per test by microscopy	9.77 per patient tested and treated
Afghanistan (societal) (2009–2012)^27^	Societal	≥ 90	10/10	Moderate transmission		
	Serv. incl.			*Pf/Pv* RDT (CareStart)		10.32 per patient tested and treated
				Microscopy		10.64 per patient tested and treated
				Low transmission		
				*Pf/Pv* RDT (CareStart)		14.66 per patient tested and treated
				Microscopy		22.38 per patient tested and treated
Tigray, Ethiopia (2006)^28^	Provider	31.5	9/10	Presumptive treatment	0.69–2.77 per course (AL)	12.80 per malaria case treated
				*Pf* RDT (Paracheck) + treatment	0.68 per test	5.38 per malaria case treated
				*Pf*/*Pv* RDT (Parascreen) + treatment	1.21 per test	7.88 per malaria case treated
Thailand, near Myanmar border (2000–2001)^29^	Societal	50	9/10	Microscopy + treatment		13.23 per true-positive case
	Serv. incl.					17.64 per *Pv* case
				ICT *Pf*/*Pv* assay + treatment	4.46 per test	10.17 per true-positive case
						14.45 per *Pv* case
				*Pf/Pv* RDT (OptiMal) + treatment	4.24 per test	8.36 per true-positive case
						6.94 per *Pv* case
Madang, East Sepik, Papua New Guinea (2005–2007)^30^	Societal	30	10/10	CQ + SP (base case)	0.03 per treatment	4.36 per *Pv* case treated
	Serv. incl.			ARTS + SP	0.43 per treatment	4.98 per *Pv* case treated
				DHA + PPQ	0.27 per treatment	4.25 per *Pv* case treated
				AL	0.29 per treatment	5.62 per *Pv* case treated
				ARTS+SP vs. CQ+SP		0.62 per *Pv* case treated
				DHA+PPQ vs. to CQ+SP		−0.11 per *Pv* case treated
				AL vs. CQ + SP		1.26 per *Pv* case treated
Rakhine State, Myanmar (1998–1999)^31^	Provider	52	9/10	EDAET (RDT + AL/[CQ+PQ])	0.30 per *Pv* treatment	1.05 per child year
	Serv. incl.				0.62 per *Pf* treatment	19 per DALY averted
					0.80 per RDT	
Amazon, Brazil (2009–2011)^32^,^33^	Provider	83	9/10	*Pv* microscopy	8.85–11.27 per test	
	Serv. incl.			CQ (3 days)	0.12 per treatment	
				CQ (3 days) + PQ (7 days)	0.23 per treatment	
				CQ + prophylaxis (12 weeks)	0.28 per treatment	
				G6PD *diagnosis:*		
				CS-G6PD vs. routine	4.14 per test	4.30 per adequately diagnosed case
				BX-G6PD vs. routine	9.81 per test	9.96 per adequately diagnosed case
				CS-G6PD vs. BX-G6PD		2.99 per adequately diagnosed case

AL = artemether–lumefantrine; ARTS = artesunate; BX = BinaxNow; CS = CareStart; CQ = chloroquine; DALY = disability-adjusted life year; DHA = dihydroartemisinin; EDAET = early diagnosis and effective treatment; G6PD = glucose-6-phosphate dehydrogenase; ICT = immunochromatographic test; *Pf* = *Plasmodium falciparum*; PPQ = piperaquine; PQ = primaquine; *Pv* = *Plasmodium vivax*; RDT = rapid diagnostic test; SP = sulfadoxine–pyrimethamine; USD = U.S. Dollar. All costs have been inflated to 2013 USD. All costs, unless otherwise indicated, are per person diagnosed or treated. Serv. incl. denotes that service delivery is included in the costing scope.

* Cost saving (dominant).

**Table 2:** Studies of the costs and cost-effectiveness of vector control interventions

Study area (year)	Perspective and scope	*Plasmodium vivax* cases (%)	Drummond's criteria	Intervention	Cost 2013 USD	Cost-effectiveness 2013 USD
Rakhine State, Myanmar (1998–1999)^31^	Provider serv. incl.	52%	9/10	ITNs vs. control	10.00 per net distributed 5.56 per person protected	51 per DALY averted
				ITNs + EDAET* vs. EDAET alone		148 per DALY averted
Thailand, villages near Myanmar border(1993–1994)^34^,^35^	Provider	20–33	9/10	Control (surveillance only)	3.43 per person at risk	
	serv. incl.			ITNs (impregnation only)	0.55 per person protected	7.89 per case averted (compared with control)
				IRS (DDT)	1.32 per person protected	16.54 per case averted (compared with control)
				ITNs (impregnation only) + control	2.28 per person protected	−16.80 per case averted*
				IRS (DDT) + control	2.80 per person protected	−7.88 per case averted*
Gujarat, India (1997–1998)^36^	Societal	NA	10/10	ITNs vs. control (EDPT†)	3.52 per net distributed	74.84 per case averted
	Serv. incl.				2.25 per person protected	
				IRS (cyclthin) vs. control (EDPT)	10.77 per house sprayed	126.39 per case averted
					2.05 per person protected	
				ITNs vs. IRS (cyclthin)		32.36 per case averted
Ninh Thuan, Vietnam forest (2012)^37^	Societal serv. incl.	47	10/10	long-lasting insecticide treated hammock vs. control	11.93 per hammock	127.85 per case averted
Colombia (2000–2001)^38^	Provider serv. incl.	NA	6/10	ITN impregnation (twice yearly)	6.71–16.32 per net impregnated	
					4.87–11.19 per person protected	No effectiveness data
				IRS (lambdacyhalothrin, twice yearly)	48.43–62.90 per house	
					9.74–13.55 per person protected	
Solomon Islands (1989–1990)^39^	Provider	NA	6/10	ITNs	3.71 per person protected	No effectiveness data
	Serv. incl.			IRS (DDT)	8.22 per person protected	
Hoa Binh, Vietnam mountain (1996)^40^	Provider	NA	6/10	ITNs (5-year duration)	1.34 per person year	
	Serv. incl.			ITN (impregnation only, twice yearly)	0.48 per person year	No effectiveness data
				IRS (lambdacyhalothrin, once yearly)	0.70 per person year	

DALY = disability-adjusted life year; DDT = dichlorodiphenyltrichloroethane; ITN = insecticide-treated bed net; IRS = indoor residual spraying; NA = not applicable. All costs have been inflated to 2013 USD. Cases refer to both cases of *Plasmodium vivax* and *Plasmodium falciparum*. Unless stated otherwise, studies were costed from a provider perspective. Serv. incl. denotes that service delivery is included in the costing scope.

* Cost saving (dominant). Early diagnosis (with RDTs) and early treatment (with ACTs).

† Early diagnosis and prompt treatment.
