# Supplementary material for: Costs and Cost-Effectiveness of Plasmodium vivax Control
Source: Am J Trop Med Hyg. 2016 Dec 28;95(6 Suppl):52–61. doi: 10.4269/ajtmh.16-0182 (PMC5201223; doi:10.4269/ajtmh.16-0182)
Supplement: Supplementary file 1 [file SD2.pdf]

Supplemental Table 1

Drummond's checklist for assessing economic evaluations

		Table 1: Diagnosis and Treatment									Table 2: Vector Control						
	Drummond's checklist for assessing economic evaluations	de Oliveira 2010	Fernando 2004	Albertini 2012	Hansen 2015	Lemma 2011	Bualombai 2003	Davis 2011	Smithuis 2013	Peixoto 2016	Kamolratanakul 2001	Butraporn 1999	Bhatia 2004	Morel 2013	Kroeger 2002	Kere 1992	Verle 1999
	Cost-effectiveness study?	1	0	0	1	1	1	1	1	1	1	1	1	1	0	0	0
1	Was a well-defined question posed in answerable form?	1	1	1	1	1	1	1	1	1	1	1	1	1	1	1	1
1.1	Did the study examine both costs and effects of the service(s) or program(s)?																
1.2	Did the study involve a comparison of alternatives?																
1.3	Was a viewpoint for the analysis stated and was the study placed in any particular decision-making context?																
2	Was a comprehensive description of the competing alternatives given (i.e., can you tell who did what to whom, where, and how often)?	1	1	0	1	1	1	1	1	1	1	1	1	1	1	1	1
2.1	Were there any important alternatives omitted?																
2.2	Was (should) a do-nothing alternative considered?																
3	Was the effectiveness of the program or services established?	1	1	NA	1	1	1	1	1	0	1	1	1	1	0	0	0
3.1	Was this done through a randomised, controlled clinical trial? If so, did the trial protocol reflect what would happen in regular practice?																
3.2	Was effectiveness established through an overview of clinical studies?																
3.3	Were observational data or assumptions used to establish effectiveness? If so, what are the potential biases in results?																
4	Were all the important and relevant costs and consequences for each alternative identified?	1	1	NA	1	1	1	1	1	1	1	1	1	1	1	1	1
4.1	Was the range wide enough for the research question at hand?																
4.2	Did it cover all relevant viewpoints? (Possible viewpoints include the community or social viewpoint, and those of patients and third-party payers. Other viewpoints may also be relevant depending upon the particular analysis.)																
4.3	Were the capital costs, as well as operating costs, included?																
5	Were costs and consequences measured accurately in appropriate physical units (e.g., hours of nursing time, number of physician visits, lost work-days, gained life years)?	1	1	1	1	1	1	1	1	1	1	1	1	1	1	1	1
5.1	Were any of the identified items omitted from measurement? If so, does this mean that they carried no weight in the subsequent analysis?																
5.2	Were there any special circumstances (e.g., joint use of resources) that made measurement difficult? Were these circumstances handled appropriately?																
6	Were the cost and consequences valued credibly?	1	1	1	1	1	1	1	1	1	1	1	1	1	1	1	1

(continued)

Supplemental Table 1

Continued

	Drummond's checklist for assessing economic evaluations	Table 1: Diagnosis and Treatment									Table 2: Vector Control						
		de Oliveira 2010	Fernando 2004	Albertini 2012	Hansen 2015	Lemma 2011	Bualombai 2003	Davis 2011	Smithuis 2013	Peixoto 2016	Kamolratanakul 2001	Butraporn 1999	Bhatia 2004	Morel 2013	Kroeger 2002	Kere 1992	Verle 1999
6.1	Were the sources of all values clearly identified? (Possible sources include market values, patient or client preferences and views, policy-makers' views and health professionals' judgements)																
6.2	Were market values used for changes involving resources gained or depleted?																
6.3	Where market values were absent (e.g., volunteer labor), or market values did not reflect actual values (such as clinic space donated at a reduced rate), were adjustments made to approximate market values?																
6.4	Was the valuation of consequences appropriate for the question posed (i.e., has the appropriate type or types of analysis—cost-effectiveness, cost-benefit, cost-utility—been selected)?																
7	Were costs and consequences adjusted for differential timing?	1	1	NA	1	NA	NA	1	0	1	1	0	1	1	0	0	1
7.1	Were costs and consequences that occur in the future "discounted" to their present values?																
7.2	Was there any justification given for the discount rate used?																
8	Was an incremental analysis of costs and consequences of alternatives performed?	1	0	NA	1	1	1	1	1	1	1	0	1	1	0	0	0
8.1	Were the additional (incremental) costs generated by one alternative over another compared with the additional effects, benefits, or utilities generated?																
9	Was allowance made for uncertainty in the estimates of costs and consequences?	1	1	NA	1	1	1	1	1	1	0	0	1	1	1	0	0
9.1	If data on costs and consequences were stochastic (randomly determined sequence of observations), were appropriate statistical analyses performed?																
9.2	If a sensitivity analysis was used, was justification provided for the range of values (or for key study parameters)?																
9.3	Were the study results sensitive to changes in the values (within the assumed range for sensitivity analysis, or within the confidence interval around the ratio of costs to consequences)?																
10	Did the presentation and discussion of study results include all issues of concern to users?	1	0	NA	1	1	1	1	1	1	1	1	1	1	1	1	1
10.1	Were the conclusions of the analysis based on some overall index or ratio of costs to consequences (e.g., cost-effectiveness ratio)? If so, was the index interpreted intelligently or in a mechanistic fashion?																

(continued)

Supplemental Table 1

Continued

Drummond's checklist for assessing economic evaluations	Table 1: Diagnosis and Treatment									Table 2: Vector Control						
	de Oliveira 2010	Fernando 2004	Albertini 2012	Hansen 2015	Lemma 2011	Bualombai 2003	Davis 2011	Smithuis 2013	Peixoto 2016	Kamolratanakul 2001	Butraporn 1999	Bhatia 2004	Morel 2013	Kroeger 2002	Kere 1992	Verle 1999
10.2 Were the results compared with those of others who have investigated the same question? If so, were allowances made for potential differences in study methodology?																
10.3 Did the study discuss the generalizability of the results to other settings and patient/client groups?																
10.4 Did the study allude to, or take account of, other important factors in the choice or decision under consideration (e.g., distribution of costs and consequences, or relevant ethical issues)?																
10.5 Did the study discuss issues of implementation, such as the feasibility of adopting the "preferred" program given existing financial or other constraints, and whether any freed resources could be redeployed to other worthwhile programs?																
Total	10	8	NA	10	9	9	10	9	9	9	7	10	10	6	6	6

Drummond's checklist for assessing economic evaluations for papers is reviewed in: White MT, Yeung S, Patouillard E, Cibulskis R. Costs and cost-effectiveness of *Plasmodium vivax* control.
